# The influence of gender and product design on farmers’ preferences for weather-indexed crop insurance

**DOI:** 10.1016/j.gloenvcha.2016.03.010

**Published:** 2016-05

**Authors:** Sonia Akter, Timothy J. Krupnik, Frederick Rossi, Fahmida Khanam

**Affiliations:** a Lee Kuan Yew School of Public Policy, National University of Singapore, 469C Bukit Timah Road, 259772, Singapore; b Social Sciences Division, International Rice Research Institute, Los Baños, Laguna 4031, Philippines; c International Maize and Wheat Improvement Center (CIMMYT), House 10/B, Road 53, Gulshan-2, Dhaka, 1213, Bangladesh

## Abstract

Theoretically, weather-index insurance is an effective risk reduction option for small-scale farmers in low income countries. Renewed policy and donor emphasis on bridging gender gaps in development also emphasizes the potential social safety net benefits that weather-index insurance could bring to women farmers who are disproportionately vulnerable to climate change risk and have low adaptive capacity. To date, no quantitative studies have experimentally explored weather-index insurance preferences through a gender lens, and little information exists regarding gender-specific preferences for (and constraints to) smallholder investment in agricultural weather-index insurance. This study responds to this gap, and advances the understanding of preference heterogeneity for weather-index insurance by analysing data collected from 433 male and female farmers living on a climate change vulnerable coastal island in Bangladesh, where an increasing number of farmers are adopting maize as a potentially remunerative, but high-risk cash crop. We implemented a choice experiment designed to investigate farmers’ valuations for, and trade-offs among, the key attributes of a hypothetical maize crop weather-index insurance program that offered different options for bundling insurance with financial saving mechanisms. Our results reveal significant insurance aversion among female farmers, irrespective of the attributes of the insurance scheme. Heterogeneity in insurance choices could however not be explained by differences in men’s and women’s risk and time preferences, or agency in making agriculturally related decisions. Rather, gendered differences in farmers’ level of trust in insurance institutions and financial literacy were the key factors driving the heterogeneous preferences observed between men and women. Efforts to fulfill gender equity mandates in climate-smart agricultural development programs that rely on weather-index insurance as a risk-abatement tool are therefore likely to require a strengthening of institutional credibility, while coupling such interventions with financial literacy programs for female farmers.

## Highlights

• We identify gender-specific needs and barriers to weather-index insurance.
• We test farmers’ preferences for non-traditional weather-index insurance-savings bundles.
• Our results indicate a gender gap in farmers’ weather-index insurance product preferences.
• This gap appears to be caused by differences in institutional trust and financial literacy.
• Bundled weather-index insurance-savings disinterest women; most farmers favor standalone inundation insurance.

## 1 Introduction

Weather related risks are major sources of income fluctuations for rural farm households in low-income countries. To buffer against such risks, and to encourage investment in intensified and high-value production, weather-index insurance (WII) is increasingly suggested for smallholder farmers (Collier et al., 2009). In a WII scheme, payouts occur when a specified weather parameter is surpassed (e.g. seasonal rainfall falls below a specified threshold indicative of drought status, or a storm passes a severity index indicating crop damage). The chosen threshold must be objective, reliably measured, and strongly positively correlated with the insured’s losses.

WII has been suggested as the “missing link” to de-risk smallholder investment in intensified cropping (Johnson, 2013), especially in the context of climate change (Collier et al., 2009). Studies have however documented a number of important limitations to WII. For example, ‘basis risk’ which arises due to poor correlation between indices and individual farmers’ experiences of crop losses, limits farmers’ initial or sustained investment in WII (Clarke and Grenham, 2013). Binswanger-Mkhize (2012) argues that most smallholder, farmers simply cannot afford to invest in insurance, while larger farmers ‘self-insure’ against risks through enterprise diversification, crop storage, and social safety nets. In some environments, farmers can self-insure against such risks through the use of more resilient crop and farm management practices. However, depending on the (opportunity) costs of these practices and the price of WII, the latter may over time crowd out the former, leading to less resilient farm management practices in the long run (Müller et al., 2011).

While these fundamental problems constraining widespread uptake remain inadequately addressed, WII projects are nonetheless operational in Africa, South and South East Asia, and Central and South America, funded by a multitude of international organizations (Binswanger-Mkhize, 2012). Despite emphasis on the business logic of WII, in practice many WII schemes remain heavily subsidized by government and donor agencies, suffering from a low rate of uptake that limits commercial viability (Cole et al., 2013). WII programs also often fail to attract the target clients most in need of protection against weather shocks, including underrepresented women farmers (Giné et al., 2008, Delavallade et al., 2015).

WII pilot programs place growing emphasis on attracting women farmers (World Bank, 2015), as women in developing countries tend to be among the poorest and the most vulnerable to climate change and weather shocks (Ahmad, 2012, Kelkar, 2009). Relative to their male counterparts, women experience gender gaps with less access to finance, inputs, education, and associated agricultural extension services. These factors consequently reduce the productivity of women’s farms by 20–30% compared to those managed by men (FAO, 2011). Eliminating gender gaps in agriculture by ensuring female farmers’ adequate and equitable access to agricultural finance, while also reducing investment risks, is thus paramount to achieving the Sustainable Development Goals to eradicate poverty and hunger, and to promote gender equality through women’s empowerment.

This paper examines male and female maize farmers’ preference heterogeneity for WII in Bangladesh, a low-lying deltaic country located at the mouth of the Bay of Bengal in the northern Indian Ocean, where interest in WII schemes is growing (Ahmed and Hasemann, 2013). In 2010, women comprised 40% of Bangladesh’s total agricultural labour force, with a 7% growth in women’s participation in agriculture between 2005 and 2010 (BBS, 2011). Women’s ability to generate income in the agricultural sector is nonetheless impeded by their low social empowerment, in addition to weak community influence and a lack of control over and access to income and resources (Sraboni et al., 2013). Women in Bangladesh are also highly vulnerable to climate change risks due to social norms, inequality and reproductive responsibilities (WEDO, 2008, Ahmad, 2012). Their capacity to adapt to climate change risks is also lower than men’s due to lack of access to financial services, limited economic opportunities, and limited voice in decision making – especially in rural areas – where only 18 percent of adult women earn an income (WEDO, 2008, Ahmad, 2012). Against this backdrop, promoting gender equality and women’s empowerment remains an important agricultural development and climate change adaptation objective for many organizations (Ahmad, 2012).

The Government of Bangladesh emphasizes diversifying and increasing crop production in the coastal region to boost national food and income security (MOA and FAO, 2013, Akter and Basher, 2014). By consequence, maize (*Zea maize*) is increasingly promoted as a supplementary cash crop (sold into Bangladesh’s burgeoning poultry and fish feed industries) within the southwestern and south central coastal zone. Relative to other field crops, optimizing maize yield generally requires increased nutrient and labor inputs. Maize has also been described as a risk-prone crop (Ali et al., 2009), because in addition to the increased financial costs of production, the long-duration of most dry season maize cultivars extends their growth period into the early monsoon season, when the risk of crop damage resulting from severe weather events increases. Promotional efforts to expand maize cultivation in coastal Bangladesh will therefore remain under-scaled if these weather associated production risks are not addressed.

The objective of the present study is to explore the ways in which gender and insurance product design influence the efficacy of inclusive WII schemes to de-risk crop production and encourage cropping intensification in the coastal south of Bangladesh, as a case study informative for similar development efforts in South Asia’s coastal zones. In order to adequately consider male and female farmers’ preferences and constraints for WII options in this risk-prone region, we conducted an attribute-based choice experiment survey in which 433 male and female maize farmers were queried on their preferences for a range of maize-based index insurance options. Preferences were modeled with regard to risk type, risk premium, payouts, trigger levels, and bundling options. The latter denotes a savings component built into the hypothetical insurance plan being offered, and represents one avenue for enhancing the value proposition of WII for potential clients. This study thus offers three contributions to the literature. First, it identifies both gender-specific needs for WII and barriers to WII demand, in a country and a region characterized by considerable climate change risks as defined by the IPCC (2012) and low women’s empowerment. Second, it tests farmers’ preferences for non-traditional WII products that are bundled with savings in an innovative way. Finally, it presents a framework for undertaking a preliminary, *ex-ante* demand oriented assessment of a WII scheme in the form of an attribute-based choice experiment, with important implications for index insuring organizations, donors, and policymakers.

## 2 Men, women, and weather index insurance

Previous studies in Bangladesh found that the market for a standard, stand-alone weather micro-insurance is characterized by low demand, poor governance, and lack of prospects for commercial viability (Akter et al., 2009, Akter et al., 2011). Akter et al. (2011) showed that these insurance schemes are likely to suffer 25% to 50% losses each year. Insurance delivery cost played a significant role in determining the commercial viability of the previously tested insurance models (Akter et al., 2009, Akter et al., 2011). The low transaction costs and reduced potential for moral hazard in WII conversely makes it an attractive alternative (Akter, 2012) and hence, interest in WII schemes is expanding in Bangladesh, particularly in the coastal districts where vulnerability to climate change is most acute (Akter and Mallick, 2013). The Asian Development Bank (ADB), for example, is planning pilot WII programs in the coastal region with national agricultural research centers (Ahmed, 2013). The International Finance Corporation has also completed a scoping study of WII investment opportunities in Bangladesh (Ahmed, 2013), and Oxfam has implemented a meso-level flood index insurance pilot program in 14 districts (Ahmed and Hasemann, 2013), though not in the coastal region.

Available evidence in India and Africa indicates that farmers’ voluntary participation in WII pilot programs has however been much more modest than anticipated by their proponents (Giné et al., 2008, Cole et al., 2013), with the uptake rate for WII among female farmers considerably lower than their male counterparts (Delavallade et al., 2015). Two mutually reinforcing problems have been observed, namely: (1) a lack of financial literacy among poorer and less formally educated farmers (Clarke and Grenham, 2013), and (2) a lack of trust in the insurance provider to deliver payouts (Carter et al., 2014, Giné et al., 2008). Additionally, risk-averse households are less likely to purchase WII as a result of uncertainty about the risk mitigation instrument that arises from their lack of experience with such products and availability of self-insurance or other alternative coping measures, provided their opportunity costs are comparatively low (Giné et al., 2008, Quaas and Baumgärtner, 2008, Baumgärtner and Quaas, 2009, Akter and Fatema, 2011).

Critics also argue that WII and other rural financial products are generally designed for men, and that they fail to account for gender-specific needs and constraints (Fletschner and Kenney, 2014). Lack of information about financial institutions and a low level of financial literacy can impede women’s ability to benefit from financial services (Cole et al., 2013). Even when women have access to information, they may fail to fully comprehend the conditions of complex financial products like WII due to their lack of confidence and exposure to the formal and official language used in most insurance contracts (Hung et al., 2012).

Men and women typically exhibit different personality traits, particularly in terms of their willingness to take risks and to trust people. In general, women tend to make less risky choices (Eckel and Grossman, 2008), and are also less likely to trust others in financial trust games (Buchan et al., 2008), although women have been shown to be more trustworthy compared to men. These phenomena are attributed to gender differences in emotional experiences of negative outcomes, especially lower utility resulting from bad outcomes experienced by women compared to men (Croson and Gneezy, 2009). Although an individual’s risk orientation is expected to be correlated with their decision to trust (e.g. risk averse individuals are less likely to place trust in an insurance provider), empirical evidence suggests that individuals do not consider trust as a problem of risk, but rather as a problem of judgment (Eckel and Wilson, 2004). The correlation between trust and risk has therefore been observed to be low and insignificant (Eckel and Wilson, 2004). The existence of a gender gap in risk preference and trust in financial decisions in particular, therefore, has different implications for men’s and women’s insurance choices. The former implies that women tend to have a stronger preference for insurance as it could help them to invest in riskier but more profitable enterprises. The later suggests that women’s tendency towards distrust may hinder their participation in non-traditional and innovative financial products like WII, especially in communities where fraudulent incidents are common.

Finally, projects seeking to improve WII tend to focus on insurance product design and quality. Bundling WII with other financial products (e.g. savings) has consequently been proposed (Carter et al., 2014, Stein and Tobacman, 2011). Bundled insurance-savings products provide a positive payment in both good and bad states of the world, making insurance clients feel that they are receiving some return on their insurance investment, even without calamity (Akter, 2012). Additionally, savings are commonly used as a form of insurance to cover against idiosyncratic shocks (such as health risks) both in developed and developing countries (Clark and Mitchell, 2014). Given that women are more vulnerable to health and environmental shocks (Clark and Mitchell, 2014), bundling WII with savings may be more suitable to women’s needs as it provides coverage against both idiosyncratic and covariate shocks (Delavallade et al., 2015). However, bundling may also potentially make the product more complicated, which could discourage women clients’ participation if they have less financial literacy than men.

## 3 Materials and Methods

### 3.1 Choice experiments

Attribute-based choice experiments (CE) are widely used for product designing and value elicitation in the absence of a real market and revealed preference data. A CE constructs a hypothetical market by presenting respondents with a series of ‘choice sets’ comprised of paired alternative plans (e.g. ‘Plan A’, ‘Plan B’). Each plan consists of the same attributes (typically three to five) that define and describe the topic of interest (e.g. a WII plan). Each attribute is defined by two or more levels, and can be represented by either a qualitative or a quantitative variable. The most notable advantage of the CE technique is that it allows attribute trade-offs and thus separately estimates the value of individual attributes of a product or program (Hanley et al., 2001). However, the multiple attributes and their levels may make the choice task complicated by imposing significant cognitive load on the respondents (Hanley et al., 2001); the number of choice sets and attributes to present to them are therefore important considerations in terms of limiting response fatigue (Caussade et al., 2005, Rossi et al., 2011). The following subsections describe the theoretical model and the structure of the CE used for our study.

### 3.2 Theoretical model

The random utility maximization (RUM) model is the underlying structural model encompassing discrete choice behaviour (McFadden, 1974). The RUM model partitions indirect utility (*U_n_*) into an observable (*V_n_*) and an unobservable, or random, component (ε*_n_*) for each respondent (*n*), as represented in Eq. (1):

(1) *U_ni_ (X_ni_)* = *V_ni_ (X_ni_)* *+* *ε_ni_*

In this model, an individual *n* faces a choice of one alternative from a finite set *C* with a vector of attributes *X*. The probability (*p*) that alternative *i* will be chosen is equal to the probability that the utility gained from its choice is greater than or equal to the utilities of choosing another alternative in *C*. Thus:

(2) $$p_{ni}=p\left\{V_{ni}+\varepsilon_{ni}\geq V_{nj}+\varepsilon_{nj};i\neq j,\forall j\in C\right\}$$

Assuming $\varepsilon_{ni}$ is identically and independently distributed and follows a Gumbel distribution, $p_{ni}$ can be estimated using the conditional logit model (McFadden, 1974), such that:

(3) $$p_{ni}=\frac{exp\left(\beta X_{ni}\right)}{\sum_{j\in C}exp\left(\beta X_{nj}\right)}$$

where β is a vector of parameter coefficients associated with *X* (i.e. the attributes).

The treatment of unobserved preference heterogeneity plays a crucial role in choice data analysis. In this study we employ the Latent Class Model (LCM) that accommodates unobserved preference heterogeneity at the group level through a discrete distribution over unobservable latent classes of respondents (Wedel and Kamakura, 2002). The LCM is typically preferred over other commonly used models, such as the Random Parameter Logit (RPL) model which treats unobserved heterogeneity using pre-specified distribution (e.g. normal, triangular, lognormal) at the individual level. The LCM is especially useful in new product development and/or targeting research because it can identify preferences and characteristics of distinct classes of respondents (cf. Birol et al., 2009).

Using the LCM, Eq. (3) is rewritten as:

(4) $$p_{ni|s}=\frac{exp\left(\beta_{s}X_{ni}\right)}{\sum_{j\in C}exp\left(\beta_{s}X_{nj}\right)}$$

where *s* stands for segments (*s* = 1, …, *S*) and *β_s_* are segment-specific utility coefficients. Now consider the following latent membership likelihood function M* that classifies individuals into one of the *S* segments:

(5) $$M_{ns}^{*}=\lambda_{s}Z_{n}+\xi_{ns}$$

where *Z* represents observed individual and household characteristics (e.g. gender, education, farm size, insurance familiarity) and $\xi_{ns}$is the error term. The probability of an individual to belong to a specific segment *s* can be described as:

(6) $$p_{ns}=\frac{\exp\left(\lambda_{s}Z_{n}\right)}{\sum_{s\in S}exp\left(\lambda_{s}Z_{n}\right)}$$

where $\lambda_{s}$ are the segment specific parameters to be estimated. Thus the unconditional probability that a randomly chosen individual *n* chooses *i* is given by Eqs. (4) and (6) by means of taking the expectation over all the *S* segments:

(7) $$p_{ni}=\sum_{s=1}^{S}\left[\frac{\exp\left(\lambda_{s}Z_{n}\right)}{\sum_{s\in S}exp\left(\lambda_{s}Z_{n}\right)}\right]\left[\frac{\exp\left(\beta_{s}X_{ni}\right)}{\sum_{j\in C}exp\left(\beta_{s}X_{nj}\right)}\right]$$

The log likelihood function maximized to estimate $\lambda_{s}$ and $\beta_{s}$ is given by:

(8) $$L=\overset{N}{\underset{n=1}{\sum }}\underset{i\in j}{\sum }\delta_{ni}\ln p_{ni}$$

where *N* is sample size and *δ_ni_* equals 1 if an individual *n* choses *i,* or 0 otherwise.

### 3.3 Choice experiment design

Four key attributes were chosen after two preliminary focus group discussions (FGDs) with male and female farmers in the study area (Table 1), including (1) *Risk Hazard Type (HAZ)*, (2) *Deposit (DEP)*, (3) *Bad Time Payment (BTP)* (payment received by the insured when a threshold surpasses an index indicative of crop damage), and (4) *Guaranteed Good Time Payment (GTP)* (payment received by the insured even if no crop damage occurred). The levels of these attributes were chosen after interviews with local government, NGO officials, extension workers, village leaders and CIMMYT scientists. The attributes and their levels were finalized after two pre-testing.

Three implicit bundling options were presented by the variable levels of GTP: *No* (*GTP* = *0*), *Partial (*0 < *GTP* *<* *Deposit*), and *Full Return (GTP* = *Deposit*). Together, *Partial* and *Full Return* represent the partial and full savings component of a bundled plan respectively, while *No Return* implies a stand-alone insurance plan. *No Return* plans contain relatively lower deposits, in contrast to the *Full Return* and *Partial Return* plans (Table 1). Technically, the interest earned from the deposit of the latter two plans pays off the insurance premium. This means that the net deposit (i.e. *DEP–GTP*) under each of these schemes were not substantially different from each other.

Based on preliminary FGD results, we represented the risk of crop damage by inundation, windstorms, and hailstorms, each being described via an associated trigger level constructed by combining two weather parameter thresholds (Appendix A in Supplementary material). Table 2 lists a set of three such composite indices constructed for each risk type. We relied upon the best-estimates of experienced agronomists to identify these trigger levels due to the absence of historical maize damage data in the study area. Each respondent was presented with only one set of trigger levels for all risk types, but different respondents were randomly shown one of the three sets of trigger levels presented in Table 2.

Although the provider is an important attribute of insurance design (Brouwer and Akter, 2010), this attribute was kept fixed to limit CE complexity (Caussade et al., 2005). Local NGOs currently operating in the study area were mentioned as the most likely provider of this insurance scheme (Appendix B in Supplementary material). This was deemed appropriate because of three reasons. First, previous research identified NGOs as the most suitable institution for insurance delivery due to their access to a large client network, infrastructural facilities across even the most remote parts of Bangladesh, a greater degree of trust and credibility among clients, and pre-existing information on client portfolios and risk history (Akter et al., 2011). Second, prospective WII pilot projects consider NGOs as the most potent insurance delivery agents due to the above mentioned factors (ADB, 2013). Finally, no evidence of distrust against the NGOs was found in the study area during the FGDs and key informant interviews.

**Fig. 1:**
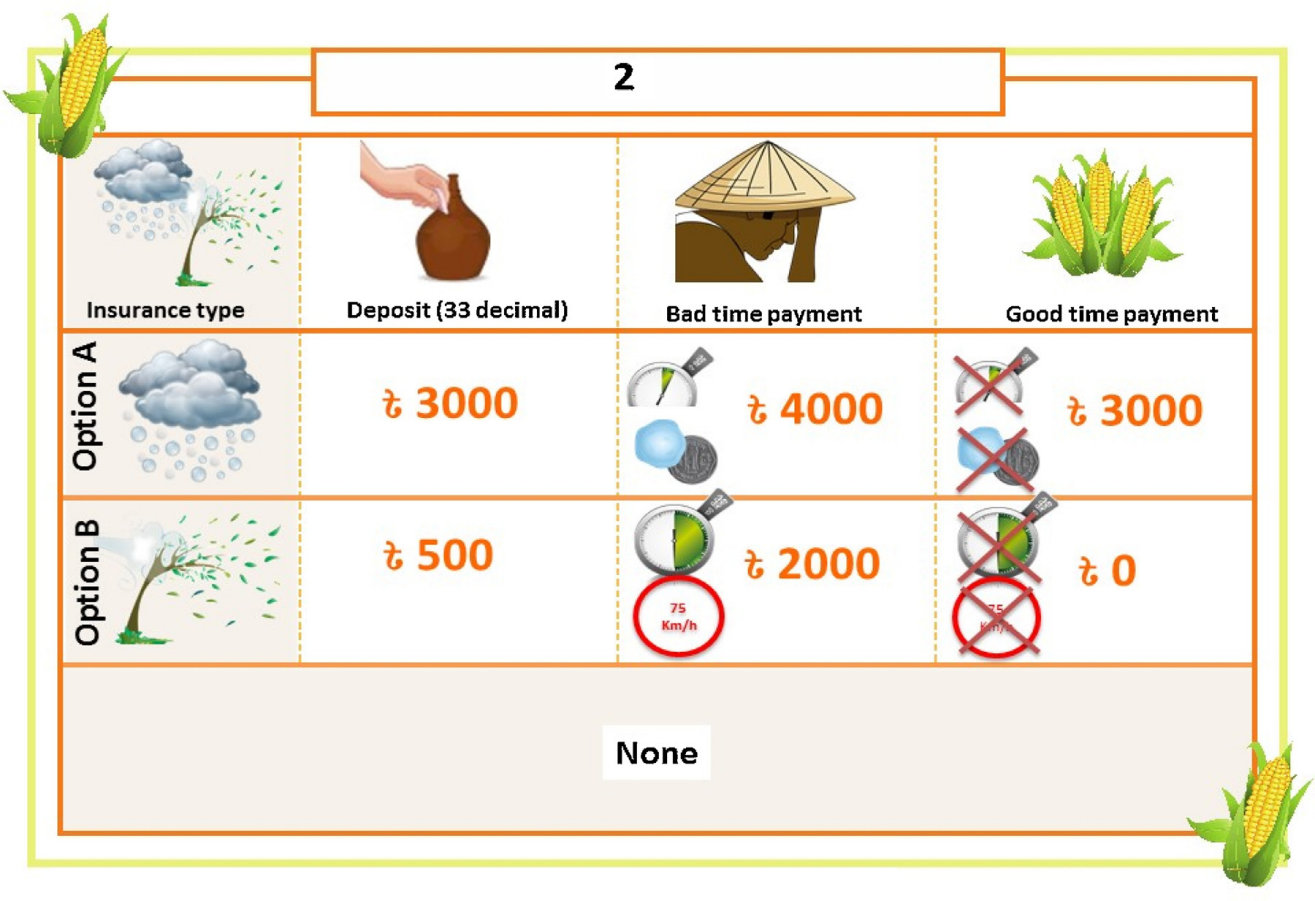
Example of a choice experiment question format shown to farmers (hail or windstorm crop damage).

Following the procedures explained by Bliemer et al. (2008) for constructing a Bayesian efficient design (‘Db-optimal efficient design’), the CE design used for this study includes 24 choice combinations randomly divided into four blocks (six choice questions in each block); thus, each respondent was randomly presented with one of the four blocks. Each choice set included two ‘unlabeled’ or ‘generic’ options, plus an opt-out alternative (‘None’) representing the *status quo* (Fig. 1). Respondents were first introduced to the hypothetical scheme through a detailed scenario description (Appendix B in Supplementary material), followed by an explanation of the corresponding trigger levels. Only then were they presented with the choice sets. Enumerators also read a ‘cheap talk’ script (Appendix C in Supplementary material) to reduce hypothetical bias (Cummings and Taylor, 1999).

### 3.4 Choice experiment implementation

**Fig. 2:**
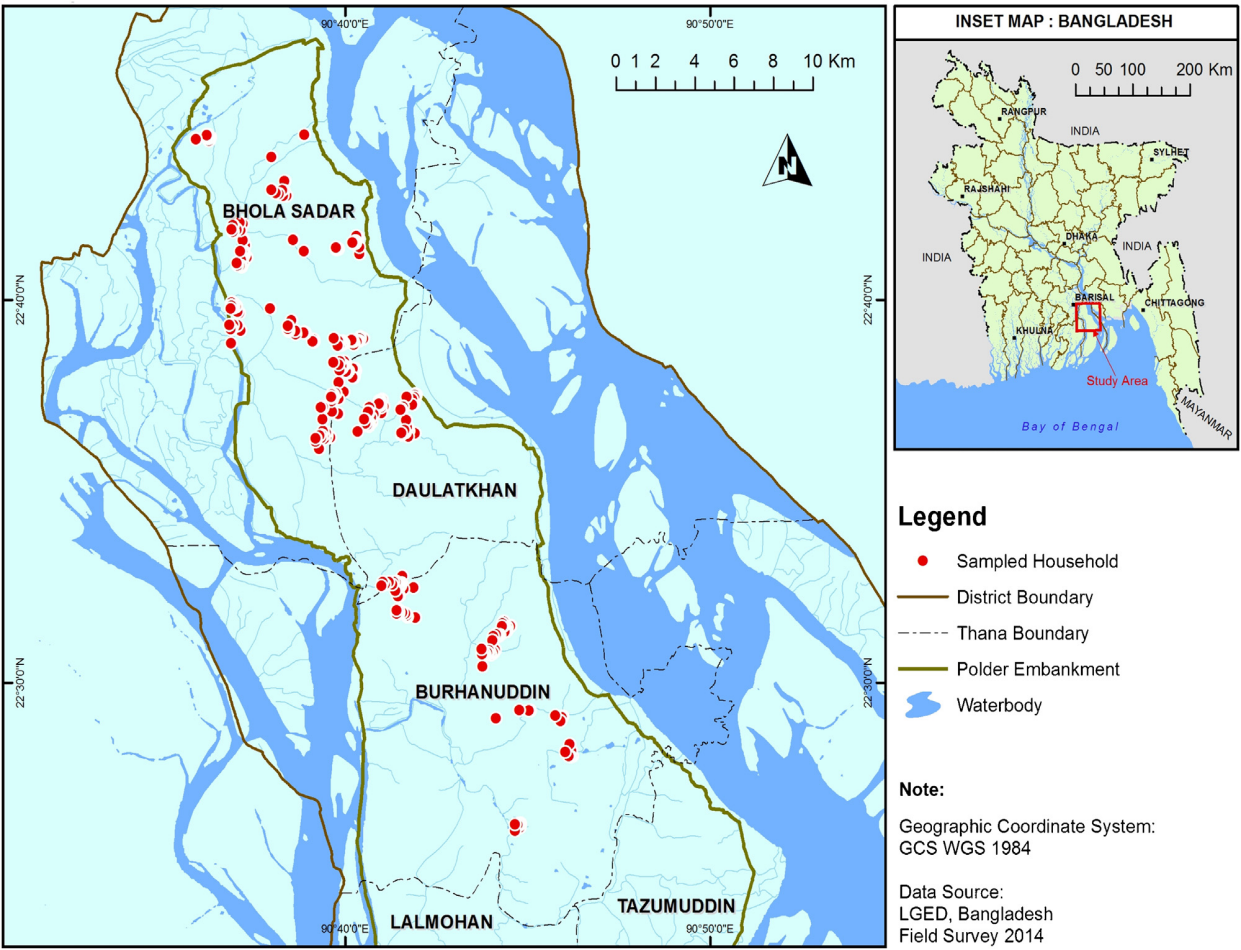
Sampled maize farming households on Bhola Island, Bangladesh.

A household survey was conducted in three sub-districts of Bhola island, namely, Bhola Sadar, Borhanuddin and Daulatkhan (Fig. 2). Maize has been promoted in these sub-districts by a large Bill and Melinda Gates Foundation and USAID-funded project (the Cereal Systems Initiative for South Asia, or CSISA), and the USAID Mission supported CSISA expansion project in Bangladesh (CSISA-BD), since the winter season of 2011–12. All listed maize farmers, male and female, in the three sub-districts were considered as the sample frame; farmer lists were obtained from the local offices of the Department of Agricultural Extension (DAE), CSISA, and local NGOs. Women farmers comprised 20% of the sample frame. In this context, ‘women farmers’ refer to women who were encouraged by extension agents to enlist themselves as farmers with the local NGO or the local DAE offices. Such enlisting helps the NGOs and DAE to fulfill their women beneficiary targets. They are not necessarily household heads, but are commonly the primary female decision makers in each household. In total, 433 fully structured face-to-face interviews (70% men and 30% women) were conducted by 20 local enumerators (14 male, 6 female, the latter primarily interviewing female respondents) between 10 and 28 June 2014 (see Appendix D in Supplementary material for more survey details). Women were slightly oversampled to reflect the standard (minimum) 30% target of reaching female beneficiaries by development projects (Bill and Melinda Gates Foundation, 2008).

### 3.5 WII model specification

For the present study, the observed component of the indirect utility function is specified as:

(9) *V* *=* *β_1_* × *ASC + β_2_ × DEP + β_3_ × BTP + β_4_* × *GTP + β_5_* × *(DEP* × *Hail) + β_6_* × *(BTP* × *Hail) + β_7_* × *(GTP* × *Hail) + β_8_* × *(DEP* × *Wind) + β_9_* × *(BTP* × *Wind) + β_10_* × *(GTP* × *Wind)*

The expression ‘*ASC*’ refers to the *alternative specific constant*, which equals zero for the status quo, and 1 for the WII plans. The *ASC* variable absorbs and isolates the (non-zero) mean utility associated with unobserved attributes of the insurance options that are not explicitly included in the CE. Two of the hazard alternatives (*Hail, Wind*) appear in the model as an interaction variable with the other principal attributes (*DEP*, *BTP, GTP*); the third alternative (*Inundation*) is the base category.

In addition to Eq. (9), a separate model is specified to test various hypotheses with respect to bundling options (*k*) and trigger levels:

(10) *V* *=* β_k_ × ASC_k_ + β_2_* × *DEP + β_3_ × BTP + β_4_* × *GTP + β_5_* × *(DEP* × *Hail) + β_6_* × *(BTP* × *Hail) + β_7_* × *(GTP* × *Hail) + β_8_* × *(DEP* × *Wind) + β_9_* × *(BTP* × *Wind) + β_10_* × *(GTP*Wind) + β_z_* × *(Z* × *ASC_k_)

In Eq. (10), *ASC* equals zero for the status quo and 1 for the bundling options (i.e. *Full*, *Partial* and *No Return*), *TRG* is a dummy variable for different trigger levels, and *Z* is a vector of socio-economic and demographic characteristics. The interaction of respondent-specific characteristics with the *ASC* in the utility function enables the identification of intra-segment heterogeneity in a LCM model.

### 3.6 Semi-quantitative follow-up study

To complement the quantitative data, a semi-qualitative study was conducted in October 2014 in which 121 farmers (72 men and 49 women) were randomly selected from the initial sample of 433 respondents. Each participant first attended a fully structured personal interview followed by a FGD. To refresh participants’ memories, the insurance scheme, bundling options, and the individual-specific choice questions from the original CE survey were revisited during the personal interview. Additionally, respondents were queried regarding their views and opinions about the proposed insurance scheme, their past experience with fraudulent incidents, and their preferred insurance service provider. A total of 15 FGDs were conducted, with eight farmers participating in each FGD, conducted separately for male and female farmers.

## 4 Results

### 4.1 Choice experiment study

#### 4.1.1 Farmer statistics

Only three percent of the sampled women did not have any active engagement in maize farming. The remaining 97% spent, on average, 19–31 person-days per season per maize cropped area, performing land preparation, crop establishment, weeding, harvesting, shelling and drying. Maize area averaged 0.13 hectares. Only 10% of the female respondents, as opposed to 74% of the male respondents, had the authority to make agricultural decisions on their own. The households represented by a woman were more likely to belong to minority religious communities (*p* < 0.01), and on average owned significantly (*p* < 0.001) smaller parcels of land and non-land assets than the households represented by men (Table 3). Although their costs of production were not significantly different, they earned significantly (*p* < 0.001) lower revenues (and thus profit), which reflects a lack of market access for the households represented by a woman. Compared to men, a significantly (*p* < 0.01) smaller proportion of women completed high school and a larger proportion of women were unfamiliar with the concept of insurance (*p* < 0.10). Women were on average significantly (*p* < 0.05) more risk averse, and had a significantly (*p* < 0.001) lower discount rate (i.e., were more patient) than men.

#### 4.1.2 Farmers’ preferences for WII

The results obtained from the LCM specification in Eqs. (9) and (10) are presented in Models 1 and 2, respectively, of Table 4. Considering the log likelihood, *pseudo R*^2^, *Bozdogan AkaikeInformationCriterion* (AIC3) and *Bayesian InformationCriterion* (BIC) (Appendix E in Supplementary material), a two-segment model was considered optimum for analysis (Andrews and Currim, 2003).

Over half of the sample (59%) belonged to the first segment; the rest (41%) belonged to Segment 2. In Model 1, the alternative specific constant for Segment 1*, or ASC_NSQ|1_*, is negative and statistically significant (*p* *<* 0.001), implying that Segment 1 respondents tended to choose the status quo. Conversely, in Segment 2, *ASC_NSQ|2_*, is positive and significant (*p* *<* 0.001), indicating that these respondents were significantly more likely to choose an insured state. Given such results, we labelled Segments 1and 2 as *Insurance Averse* and *Insurance Favoured*, respectively.

In both segments of Model 1, the coefficients of *DEP*, *GTP* and *BTP* are significant (*p* *<* 0.001); the signs of the coefficients of *DEP*, *GTP* and *BTP* conform to *a priori* theoretical expectations of a lower demand for inundation based WII (the baseline alternative) due to a higher deposit requirement and higher demand due to higher good and bad time payments. In Segment 1 (Model 1), the coefficients of the interaction terms between *DEP*, *BTP*, *GTP* and *Hail* and *Wind* indicate significant preference heterogeneity with respect to hazard type among the *Insurance Averse* respondents. For *Hail* based WII, clearly, the demand is significantly lower than *Inundation* based WII. For *Wind* based WII, the net change in utility from the *Inundation* based WII was unclear at this stage (implicit price estimates in Section 4.1.3 provide clarification). In Segment 2, conversely, the coefficients of the interaction terms between *DEP*, *BTP*, *GTP* and *Hail* and *Wind* are not statistically different than zero, implying that the members of the *Insurance Favored* group were equally likely to choose *Inundation*, *Hail* and *Wind* based WII.

The segment membership coefficients suggest that female respondents were significantly (*p* *<* 0.001) more likely to be *Insurance Averse* than the male respondents. Risk preference was an important determinant of segment membership (*p* *<* 0.10), with a positive sign which conforms to previous empirical evidence that risk averse individuals are more likely to be insurance averse (cf. Giné et al., 2008). In addition, relatively wealthier households, and households that maintained a savings account with a formal institution (an indicator of financial literacy), were significantly (*p* < 0.05 and <0.01, respectively) more likely to belong to the *Insurance Favoured* group. The coefficients of all of the other variables (e.g. spouses’ presence, time preference, insurance familiarity) were statistically insignificant.

Model 2 of Table 4 decomposes insurance preference according to bundling options, controlling for trigger levels and respondents’ socio-economic and demographic characteristics. For the *Insurance Averse* group, respondents were significantly less likely (*p* *<* 0.001) to choose a *Full Return* scheme as opposed to the *No* and *Partial Return* schemes and the status quo. For the *Insurance Favoured* group, the coefficients of the *No*, *Partial, and Full Return* schemes are positive and statistically significant (*p* < 0.01 and 0.10, respectively) with the utility parameter associated with the *No-Return* scheme substantially, although not significantly, higher than the rest. These findings imply that the *Full Return* scheme was the least popular option among the *Insurance Averse* group, and that the *No Return* scheme was the most popular among the *Insurance Favoured* group, respectively. Trigger levels did not influence insurance choice in Segment 1. The *Insurance Favoured* respondents who were shown *Trigger* 2 and 3 (comprising relatively lower weather parameter thresholds than *Trigger* 1), were significantly more likely to pay a higher net deposit compared to respondents who were shown *Trigger* 1.

In the *Insurance Averse* group, women disliked the *Full Return* scheme as much as men, but conversely disliked the *Partial* and *No Return* schemes significantly more than men. In the *Insurance Favoured* group, compared to men, women were significantly less likely to opt for the *Full* and *Partial Return* schemes. No significant difference was observed between men and women in terms their preference for the *No Return* scheme in the *Insurance Favoured* group. Among other factors, risk-averse individuals in the *Insurance Averse* group were significantly (*p* *<* 0.001) more likely to choose the *Full Return* option. Time preference was not a significant determinant of intra-group preference heterogeneity.

#### 4.1.3 Implicit prices

The general formula for calculating marginal willingness to pay, or implicit price (IP), of a given attribute from a choice experiment is IP = −(*β_x_*/*β_y_*), where *β_x_* is the coefficient of attribute *x* and *β_y_* is the coefficient of the variable representing the payment vehicle. Using the parameter estimates for Model 1 (Eq. (9)) shown in Table 4, we estimated implicit prices for the hypothetical WII, and for each hazard (Table 5). IP_BTP_ in Table 5 refers to the mean willingness to pay for a scheme that offers zero “good time payment”, but conversely with 1000 Taka (∼$13) compensation following a “bad time” event. The *Insurance Averse* group’s willingness to pay for a standalone *Inundation* based WII was $2, significantly (*p* *<* 0.001) different than zero, while this group’s willingness to pay for standalone *Hail* and *Wind* based WII was considerably lower than the inundation based WII, and not significantly different than zero. Conversely, the *Insurance Favoured* group had positive and significantly (*p* *<* 0.001) higher willingness to pay for standalone insurance for all hazard based WII than that of *Insurance Averse* group. The implicit prices for different hazard based WII among the *Insurance Favoured* group were not significantly different from each other.

When a “guaranteed good time payment” or a savings component of 1,000 Taka (∼$13) is added to the WII, the implicit prices unequivocally increase for both groups and all hazard types (i.e. IP_GTP_ in Table 5). Interestingly, for the *Insurance Averse* group, the total WTP (i.e. IP_Total_ in Table 5) for a WII that offers $13 payment both in good and bad states of the world was significantly (*p* *<* 0.001) lower than $13. This implies that this group not only wants their full deposit to be returned, but also expected a positive return from their savings. This finding exhibits the *Insurance Averse* group’s willingness to accept compensation for paying a higher deposit, which is consistent with the negative sign of the utility parameter associated with *Full Return* scheme in Segment 1 of Model 2 (Table 4). The *Insurance Favoured* group’s total willingness to pay to receive $13 payment in both good and bad states of the world was not significantly different than $13, implying that they are willing to pay roughly at least $13 to receive the same amount in both good and bad states of the world.

### 4.2 Semi-qualitative follow-up study results

One hypothesis for low insurance demand in the study area was that a lack of trust in the insurance institution limits investments. Re-sampled farmers were therefore asked specific questions about their past experiences with financial fraud during the follow-up study. About a third of the respondents had been victims of financial fraud in the past (Table 6). One widely mentioned case of fraud involved accusations against a small organization, no longer active in Bhola, that offered a high return savings scheme. The alleged organization vacated Bhola after collecting money from their clients for two years without making any payouts. No significant difference was observed between men and women in terms of their likelihood of experiencing fraudulent incidents (Chi^2^ *= *1.5; *p *= 0.21). Interestingly, 10 out of the 37 fraud victims who admitted that their prior fraud experience negatively influenced their decisions during the CE survey were women. These women also tended to be more likely (*p* = 0.15) to choose the status quo during the CE survey (72% of the cases), when compared to the remaining eight female fraud victims who stated their decisions were not influenced by their past experience of fraud (58% of the cases). To test whether the experience of fraud made people more or less risk averse, we compared the risk coefficients of the male and female victims and non-victims (Table 7). The average risk coefficients of respondents whose insurance choice was affected by fraud were not significantly different *(p *= 0.42), suggesting that a lack of trust in insurance was not particularly motivated by risk aversion. This finding is consistent with previous empirical evidence regarding low and insignificant correlation between trust and risk as shown by Eckel and Wilson (2004).

This lack of institutional trust raises the question of who a preferred and trusted insurance provider could be. Most respondents (44%) stated they would place their trust in a government bank followed by NGOs (40%). This finding suggests no specific bias against NGOs, which were referred as the most likely insurance providers during the CE survey. Islamic banks were the third most preferred insurance provider, voted by 15% of the respondents, while private insurance companies were the least preferred option. No significant differences were observed between men and women, or victims and non-victims, in terms of insurance provider preferences. However, women who stated that their choices were affected by a previous fraudulent incident predominantly opted for the government bank as their most preferred insurance provider.

Low insurance demand was also influenced by aspects of the insurance product which were not explicitly captured by the CE attributes. Most participants – men in particular – stated that they did not like the core principle of WII, *i.e.* that compensation payout is linked with one or multiple weather parameters that are difficult to measure or understand from the farmer’s perspective. They expressed concern about the type of methodology used, and what could happen if the methodological approach failed to measure the parameters accurately. Farmers also feared that such products were likely to be associated with basis risk. For example, if a strong wind blows for less than 30 minutes, which was considered to be of low risk to crop damage by local agronomists, farmers nonetheless thought they could still suffer considerable damage due to maize lodging or stem breakage, although under this circumstance they would not receive a payout. Instead, they preferred the traditional insurance scheme where the payout depends on actual incurred and directly quantified damage, with damage assessment achieved through physical verification by the insurer. When given a choice between WII versus traditional insurance, 55% of the respondents, with no significant difference across men and women, chose traditional insurance over WII.

Finally, low insurance demand was also affected by low financial literacy. Both male (25%) and female (75%) respondents found the conditions of the proposed WII schemes to be complicated. In particular, female farmers struggled the most to comprehend the trigger level and compensation. Most women (75%) implied that since they are not highly active outside the household, and because they lacked higher education, they customarily rely on male household members to make financial decisions. Although in some cases male family members were present during the CE interviews, and thus occasionally helped women understand WII, a lack of self-confidence was still clearly evident and most women farmers still found the concept to be abstract and complex.

## 5 Discussion and Conclusions

Policy and donor investment prioritization efforts in Bangladesh focus attention on climate change adaptation and gender equitable agricultural development in the coastal region (MOA and FAO, 2013). Interest in the ways in which WII programs could help achieve these goals is consequently growing, with a number of preliminary feasibility studies underway by donor, research, and investment agencies (Ahmed and Hasemann, 2013, Ahmed, 2013, ADB, 2013). None of these efforts however explicitly address the potential for gender differences in smallholders’ preferences for WII, instead relying on the *a priori* supposition that WII will be equally accepted by men and women farmers, in line with suggestions elsewhere (cf. World Bank, 2015).

In response to this problem, our CE survey results showed evidence of a substantial gender gap in WII demand, with important implications for the promotion of WII programs in agricultural development and climate change adaptation efforts. Although women were significantly more risk averse, supporting Eckel and Grossman, 2002, Eckel and Grossman, 2008, and had a lower discount rate, these factors could not fully explain the gender gap. A follow-up study designed with a sub-sample of the original choice experiment participants revealed important differences across gender with respect to institutional trust, helping to qualitatively explain the gender difference in WII choice to some extent. Although women were not more or less susceptible to fall prey to financial frauds than men, women who were previous victims of financial fraud were more likely to be skeptical about the credibility of the proposed insurance scheme in delivering payouts than were men, despite similar experience. This finding can be partly explained in light of the theories of the financial economics literature which suggest women tend to feel more regretful than men due to poorly made financial decisions in the past, and thus exhibit more loss aversion behavior when it comes to making future investment decisions (Arora and Kumari, 2015). The women fraud victims further affirmed a strong preference for government banks as their preferred insurance provider during the follow-up study, supporting previous assertions of the importance of assuring institutional credibility as a precondition for WII adoption, particularly in environments characterized by poor governance and weak institutional accountability (Carter et al., 2014, Giné et al., 2008).

The follow-up study results also suggested that both men and women found WII to be conceptually complex. While women were significantly less familiar with insurance (indicative of a low level of financial literacy), men were also relatively unfamiliar–although they understood the mechanism following adequate briefing by enumerators. Most women respondents struggled to comprehend the trigger levels and compensation mechanisms in particular. This implies that, due to their relative lack of experience and exposure to financial matters, women generally failed to understand the formal language commonly used to describe such insurance schemes. This conclusion should however be carefully interpreted due to the time difference between the initial CE and follow-up study, although our efforts to brief each follow-up study participant by providing a summary of the initial experiment and their chosen insurance preferences should have minimized any inconsistency. Further research is needed to test the ways in which proposed WII schemes are designed and communicated, as our results suggest that they should be simple as possible for the intended clients – particularly women – to understand and enroll in.

We tested the potential of an innovative insurance-savings component to boost farmers’ interest in, and acceptance of WII, for women in particular through the CE survey. A “full return” based WII-savings bundle was designed to pay back the insurance premium to the purchaser, with the interest earned on the premium covering the insurer’s cost. Such a plan required a high premium deposit, however, since this is the only way to assure financial viability. This “full return” plan was the least popular option among the *Insurance Averse* group, because the initial deposit required was substantially higher than the alternative plans. There was no evidence to suggest that women preferred “full return” plans more than men. Even within the members of the *Insurance Favoured* group, who were in general supportive of all the bundling options, women were particularly averse to the bundled WII-savings schemes. Hence, our findings suggest that overall there is likely little promise for a bundled WII-savings product to attract women clients in the study area, and that a standalone insurance plan holds the greatest prospects of survival, supporting previous study in Ahmedabad, India examining the potential for rainfall WII (cf. Stein and Tobacman, 2011).

Of all weather hazard options, most CE survey participants preferred protection against inundation, suggesting that additional work on WII targeted for Bangladesh’s coastal region should address this risk in particular. The problem with the wind and hail plans tested in this study is that these events can be quite localized. Any relatively accurate (and potentially trusted) measurement of these parameters would require both accurate weather stations and their placement at an appropriate network density to detect locally specific wind events within the farm landscape in which a proposed WII scheme would be deployed. Unfortunately, the Bangladesh Meteorological Department (BMD) currently lacks such a sufficiently dense network of observation points, though efforts to increase the density of observation stations are currently underway (ADB, 2013, Ahmed and Hasemann, 2013). While additional research is needed to verify that skepticism of the accuracy of meteorological reporting is why some of our respondents did not favor wind or hail-based WII, the findings of the follow-up study indicate that (at least for males) farmers’ choices were influenced by their concerns about basis risk and also by a lack of conceptual clarity on how weather parameters could be measured. In contrast, inundation-based WII may be a more promising option because the spatial extent of flooding and consequent inundation is typically wider than hail or wind damage, and linking inundation to a trigger (water level) is more direct, and there are more river gauges than weather stations in Bangladesh.

Nevertheless, several obstacles to developing such an inundation-based WII remain in terms of their underlying technical aspects (e.g. spatial mapping via river gauge data interpolation, or remote sensing to determine flood depth and duration, or a combination of both (cf. de Leeu et al., 2014). The study area is also susceptible to excessive rain events which may cause waterlogging damage in low-elevation areas independent of riverine floods (and hence which may be poorly monitored by river gauges); thus, a significant source of basis risk remains to be addressed before inundation-based WII schemes can be effectively deployed.

Further research is required to determine how much of the observed low demand is related directly to gender, the WII product itself (e.g. lack of conceptual understanding, complexity of the WII plans, lack of trust in the implementing agency), and/or to the method used to test it (e.g. unfamiliarity with, and/or complexity of the choice experiment format). Nonetheless, it is clear that the development of WII schemes deployed in this and similar risk-prone coastal environments should consider greater emphasis on assuring and strengthening the creditability of insuring institutions, particularly as institutional trust levels vary between men and women, while also undertaking efforts to strengthen women’s financial literacy, in order to more equitably reach women beneficiaries.

## 6 Dedication and acknowledgements

This paper is dedicated to the memory of Dr. Paula Kantor who worked widely in Bangladesh and Afghanistan researching the intersection of agricultural development and gender. Dr. Kantor’s life was tragically lost in a terrorist attack in Afghanistan in 2015 and represents a significant loss to scientists working in these fields. We gratefully acknowledge the ﬁnancial support of the Yale Savings and Payments Research Fund at Innovations for Poverty Action (IPA), sponsored by a grant from the Bill & Melinda Gates Foundation (BMGF). We thank Dr. Sommarat Chantarat for her contribution during the preliminary stage of this research, and Dr. Samina Yasmin for assistance in coordinating fieldwork. We also thank Mohammad Ruhul Amin for his support in GIS, and the three anonymous reviewers for their comments that improved the manuscript. Part of this research was made possible by support from the USAID Mission in Bangladesh through the Cereal Systems Initiative for South Asia in Bangladesh (CSISA-BD) project, as well as support from the Global Rice Science Partnership (GRiSP) program under the Consultative Group on International Agricultural Research (CGIAR), and Phase II of the Cereal Systems Initiative for South Asia, funded by USAID and the BMGF. The content and opinions resulting from this research are those of the authors and do not necessarily reflect the views of IPA, BMGF, USAID, or the United States Government, and shall not be used for advertising or product endorsement purposes; nor do they reflect positions or policies of the aforementioned organizations.

## Figures and Tables

**Table 1:** Choice experiment attributes and their associated levels (all monetary values are presented in Bangladesh taka, Tk^a^).

Bundling options	Attributes	Levels
No Return	Hazard	Inundation, Windstorm, Hailstorm
Deposit^b^	100, 200, 300, 500, 800, 1000
Guaranteed good time payment	0
Bad time payment	1000, 1500, 2000, 3000, 5000

Partial Return	Hazard	Inundation, Windstorm, Hailstorm
Deposit^c^	500, 800, 1000, 2000, 2500, 3000
Guaranteed good time payment	200, 800, 1800, 2000, 2500, 2800
Bad time payment	2000, 3000, 4000, 5000

Full Return	Hazard	Inundation, Windstorm, Hailstorm
Deposit^d^	800, 1500, 2000, 2500, 3000, 4000
Guaranteed good time payment	1500, 2000, 2500, 3000, 4000
Bad time payment	1800, 2000, 2500, 3000, 3500, 4000, 5000

*Note*: Nominal interest rate on a general savings account in Bangladesh varies between 6 and 9% (Bangladesh Bank, 2015).

a Tk 77 = 1 USD.

b Net deposit (i.e. deposit–good time payment) = 100, 200, 300, 500, 800, 1000.

c Net deposit = 100, 200, 300, 500, 600.

d Net deposit = 0.

**Table 2:** Index insurance trigger levels (intensity + duration) for three types of weather related risks to maize production.

Trigger	Inundation	Windstorm	Hailstorm^a^
Level 1	Intensity = 15 cm Duration = 3 days	Intensity = 75 km/h Duration = 30 min	Intensity = 1 Tk coin Duration = 5 min
Level 2	Intensity = 15 cm Duration = 2 days	Intensity = 75 km/h Duration = 15 min	Intensity = 1 Tk coin Duration = 3 min
Level 3	Intensity = 10 cm Duration = 3 days	Intensity = 60 km/h Duration = 30 min	Intensity = any size Duration = 5 min

a Hail size circumference was compared to the sphere equivalent of a 1 Bangladesh taka coin measuring 25 mm diameter.

**Table 3:** Description of the sampled households and respondents.

Variable	Male (*n* = 299)	Female (*n* = 134)	Diff.	Z or χ^2^ value	*P*
*Household characteristics*					
Religion (non-Muslim) (%)	1%	7%	6%	9.19	<0.01
Household size	6.58	5.57	1	4.13	<0.001
Cultivable land (decimal)	103	45	58	4.67	<0.001
Non-land asset (in US$)	1973	1245	728	3.28	<0.001
Average size of maize farm in decimal (hectare)	31 (0.14)	26 (0.11)	4.72	1.17	0.30
Cost (median) of production of per hectare of maize farm in 2014 (in USD)	476	487	11	20478^a^	0.71
Revenue (median) earned from per hectare of maize farm in 2014 (in USD)	1191	1016	175	16189^a^	<0.001
Profit (median) earned from per hectare of maize farm in 2014 (in USD)	667	476	190	15925^a^	<0.001
Maize cultivation experience (years)	3.50	3.0	0.47	2.50	<0.05
Formal savings account (%)	43	49	−6	1.40	0.30
Formal credit account (%)	42	54	−12	4.72	<0.05
Purchased insurance (%)	21	22	−1	0.018	0.88

*Respondent characteristics*					
Mean age (years)	45	35	9.5	7.40	<0.001
High school and above (%)	35	22	13	7.33	<0.01
Head of the household (%)	87	23	64	171	<0.001
No familiarity with insurance (%)	53	63	−10	3.70	<0.10
Risk aversion coefficient^b^	0.73	0.91	−0.23	2.45	<0.05
Time preference^c^ (% with discount rate >70%)	86	68	18	21	<0.001

a Mann-Whitney *U* statistics.

b Assuming constant relative risk aversion (CRRA), $u\left(y\right)=y^{1-\theta }/1-\theta$, the curvature of the utility function *θ* represents the degree of risk aversion. This was determined by calculating the value of θ that would make a respondent indifferent between the chosen gamble and the two adjacent gambles (Eckel and Grossman, 2008). The mean of the risk-aversion coefficient is 0.78, which is consistent with CRRA risk-aversion coefficients for farmers in developing countries (Olbrich et al., 2012).

c The discount rate is determined solving the value function $v\left(M_{0}\right)=\frac{1}{1+r}v\left(M_{t}\right)$. *M*_0_ is the present value of *M_t_* offered at time *t* with discount rate *r*.

**Table 4:** Latent class logit model regression results.

Variables	Description		Model 1		Model 2	
			Segment 1 (Insurance averse)	Segment 2 (Insurance favored)	Segment 1 (Insurance averse)	Segment 2 (Insurance favored)
			*Mean of utility parameters (Parentheses indicate SE)*			
ASC	Alternative specific constant. Choice of an insured state = 1, otherwise = 0		−0.53*** (0.15)	0.99*** (0.18)	–	–
Full-Return	Choice of a Full-Return scheme = 1, otherwise = 0		–	–	−2.98*** (0.61)	0.91* (0.54)
Partial-Return	Choice of a Partial-Return scheme = 1, otherwise = 0		–	–	0.03 (0.55)	1.02* (0.55)
No-Return	Choice of a No-Return scheme = 1, otherwise = 0		–	–	0.28 (0.38)	1.22*** (0.41)
$\beta_{2}$ (DEP a)	Deposit		−0.005*** (0.001)	−0.002** (0.0008)	−0.006*** (0.001)	−0.003*** (0.0009)
$\beta_{3}$ (BTP a)	Bad time payment		0.0007*** (0.0002)	0.0005*** (0.0002)	0.0006*** (0.0002)	0.0005** (0.0002)
$\beta_{4}$ (GTP a)	Good time payment		0.0035*** (0.0006)	0.0017*** (0.0007)	0.005*** (0.001)	0.003*** (0.0007)
(Hail × DEP) $\beta_{5}$	Interaction between hailstorm insurance and deposit		0.0022 (0.0015)	−0.0003 (0.0013)	0.0003* (0.0002)	−0.0004 (0.001)
(Hail × BTP) $\beta_{6}$	Interaction between hailstorm insurance and bad time payment		−0.0008** (0.0004)	0.00015 (0.0003)	−0.001** (0.0004)	0.0004 (0.0003)
(Hail × GTP) $\beta_{7}$	Interaction between hailstorm insurance and good time payment		−0.0015 (0.001)	−0.57E-04 (0.001)	0.002* (0.001)	3.24E-04 (0.001)
(Wind × DEP) $\beta_{8}$	Interaction between wind insurance and deposit		0.0026*** (0.001)	0.001 (0.001)	0.003*** (0.001)	0.001 (0.001)
(Wind × BTP) $\beta_{9}$	Interaction between wind insurance and bad time payment		−0.0006*** (0.0002)	−0.0002 (0.0002)	−0.0005** (0.0002)	−0.0002 (0.0002)
(Wind × GTP) $\beta_{10}$	Interaction between wind insurance and good time payment		−0.002** (0.0008)	−0.001 (0.001)	−0.002** (0.0008)	−0.001 (0.0007)
TRG2^b^ × (DEP-GTP)	Interaction between trigger level 2 and net deposit		–	–	0.0003 (0.0005)	0.001** (0.0005)
TRG3^b^ × (DEP-GTP)	Interaction between trigger level 3 and net deposit		–	–	−0.0001 (0.0005)	0.0015*** (0.0005)

*Intra-segment (conditional) heterogeneity (Model 2 only)*						
Time × Full-Return	Interaction between time preference (Discount rate > 70% = 1) and full return urn scheme				0.42 (0.41)	0.60 (0.38)
Time × Partial-Return	Interaction between time preference (Discount rate > 70% = 1) and partial return scheme				−0.22 (0.37)	0.61 (0.38)
Time × No-Return	Interaction between time preference (Discount rate > 70% = 1) and no return scheme				−0.24 (0.35)	0.03 (0.30)
Risk × Full-Return	Interaction between risk coefficient and full return scheme				0.42*** (0.15)	−0.12 (0.17)
Risk × Partial-Return	Interaction between risk coefficient and partial return scheme				−0.04 (0.16)	−0.12 (0.18)
Risk × No-Return	Interaction between risk coefficient and no return scheme				0.02 (0.10)	−0.08 (0.15)
Female × Full-Return	Interaction between female and full return scheme				−0.50 (0.30)	−1.08** (0.45)
Female × Partial-Return	Interaction between female and partial return scheme				−0.75** (0.31)	−1.75*** (0.46)
Female × No-Return	Interaction between female and no return scheme				−0.72** (0.29)	−0.37 (0.36)

*Segment probability model*^c^						
Familiarity	Respondent is familiar with insurance = 1, otherwise = 0		0.38 (0.32)	0.0	0.43 (0.31)	0.0
Female	Female = 1, otherwise = 0		2.10*** (0.41)	0.0	0.90* (0.47)	0.0
Age	Respondent’s age (in years)		−0.002 (0.01)	0.0	−0.003 (0.01)	0.0
Literacy	Respondent has some literacy = 1, otherwise = 0		−0.28 (0.36)	0.0	−0.34 (0.34)	0.0
Risk preference	Coefficient of risk aversion		0.30* (0.17)	0.0	0.33* (0.17)	0.0
Time preference	Discount rate (>70%) = 1, otherwise = 0		−0.09 (0.37)	0.0	0.45 (0.44)	0.0
Purchased insurance	Respondents purchased insurance = 1, otherwise = 0		−0.17 (0.37)	0.0	0.06 (0.37)	0.0
Formal savings	Respondent maintains a savings account with a formal institution = 1, otherwise = 0		−1.20*** (0.30)	0.0	−1.04*** (0.30)	0.0
Credit	Respondent borrowed money from a formal institution = 1, otherwise = 0		0.20 (0.27)	0.0	0.12 (0.27)	0.0
Spouse	Spouse was present during the interview = 1, otherwise = 0		0.21 (0.36)	0.0	0.13 (0.36)	0.0
Profit	Profit earned from 33 decimal of maize in 2014 (in thousand Tk)		0.02 (0.02)	0.0	0.02 (0.016)	0.0
Asset	Value of non-land asset owned by the household (in thousand Tk)		−0.003** (0.001)	0.0	−0.002** (0.001)	0.0
Constant			0.16 (0.80)	0.0	−0.31 (0.80)	0.0
Segment probability			0.59	0.41	0.55	0.45

*Model statistics*						
Group number			433		433	
Log likelihood			−2368		−2324	
LR χ^2^			919 (df = 33, *p* < 0.0001)		1007 (df = 59, *p* <0.0001)	
McFadden Pseudo *R*^2^			0.16		0.18	

* *p* < 0.10.

** *p *< 0.05.

*** *p* < 0.01.

a Base category = Inundation.

b Base category = Trigger level 1.

c The membership coefficients for Segment 2 were normalized to zero.

**Table 5:** Mean implicit prices (IP)^a^ for weather index insurance in US$/season/bigha^b^, by weather hazard and segment (95% confidence interval in the parenthesis^c^).

Model 2, Segment 1 (Insurance Averse)				Model 2, Segment 2 (Insurance Favored)		
Hazard	IP_BTP_	IP_GTP_	IP_Total_ (=IP_BTP_ + IP_GTP_)	IP_BTP_	IP_GTP_	IP_Total_ (=IP_BTP_ + IP_GTP_)
Flood	2.00^d^ (1.50–2.50)	9.65^e^ (8.97–10.34)	11.64 (11.17–12.10)	3.12^j^ (1.87–4.38)	10.58^k^ (8.95−12.21)	13.70 (12.12–15.28)
Hail	−0.30^f^ (−4.01–3.43)	10.48^g^ (8.16–12.68)	10.19 (8.30–12.23)	3.60^l^ (2.81–4.36)	9.00^m^ (8.12–9.90)	12.58 (12.00–13.15)
Wind	0.83^h^ (−0.60–2.26)	10.15^i^ (8.95–11.34)	11.00 (9.84–12.12)	3.85^n^ (1.94−5.76)	9.30^o^ (7.33−11.26)	13.15 (11.00–15.33)

a Implicit prices for Taka 1,000 (US$13) worth of remuneration either as compensation for a “bad time” event or for savings returned during “good times”, and for both combined.

b One *bigha* = 0.134 ha. = 0.33 acre.

c Confidence intervals were estimated using the Wald procedure (Delta Method).

d $-\frac{\beta_{3|1}}{\beta_{2|1}}$.

e $-\frac{\beta_{4|1}}{\beta_{2|1}}$.

f $-\frac{\left(\beta_{3|1}+\beta_{6|1}\right)}{\left(\beta_{2|1}+\beta_{5|1}\right)}$.

g $-\frac{\left(\beta_{4|1}+\beta_{7|1}\right)}{\left(\beta_{2|1}+\beta_{5|1}\right)}$.

h $-\frac{\left(\beta_{3|1}+\beta_{9|1}\right)}{\left(\beta_{2|1}+\beta_{8|1}\right)}$.

i $-\frac{\left(\beta_{4|1}+\beta_{10|1}\right)}{\left(\beta_{2|1}+\beta_{8|1}\right)}$.

j $-\frac{\beta_{3|2}}{\beta_{2|2}}$.

k $-\frac{\beta_{4|2}}{\beta_{2|2}}$.

l $-\frac{\left(\beta_{3|2}+\beta_{6|2}\right)}{\left(\beta_{2|2}+\beta_{5|2}\right)}$.

m $-\frac{\left(\beta_{4|2}+\beta_{7|2}\right)}{\left(\beta_{2|2}+\beta_{5|2}\right)}$.

n $-\frac{\left(\beta_{3|2}+\beta_{9|2}\right)}{\left(\beta_{2|2}+\beta_{8|2}\right)}$.

o $-\frac{\left(\beta_{4|2}+\beta_{10|2}\right)}{\left(\beta_{2|2}+\beta_{8|2}\right)}$.

**Table 6:** Farmers’ experiences of fraud and insurance choice.

	Within Male (%)	Within Female (%)	χ^2^ value
Experienced fraud	27	38	1.54 (*p* < 0.25)
Fraud victims’ frequency of status quo choice	36	66	–
Non-victims’ frequency of status quo choice	34	65	–
χ^2^ value (*p* < 0.001)	0.10 (*p* <0.80)	0.01 (*p* <0.90)	
Experience of fraud impacted choice	0	56	9 (*p* <0.001)
Experience of fraud impacted choice and % of status quo choice	–	72	–
Experience of fraud did not impact choice and % of status quo choice	36	58	7 (*p* < 0.001)
χ^2^ value	–	2.105 (*p* < 0.15)	

**Table 7:** Experience of fraud and risk preference.

Risk preference coefficients			
	Experienced fraud	Did not experience fraud	Z value
Male	0.97	0.83	0.54 (*p* < 0.60)
Female	0.56	1.00	2.3 (*p* < 0.05)

	Fraud exp. impacted choice	Fraud exp. did not impact choice	Z value
Male	–	0.97	–
Female	0.59	0.52	0.66 (*p* = 0.60)
