# Supplementary material for: The influence of gender and product design on farmers’ preferences for weather-indexed crop insurance
Source: Glob Environ Change. 2016 May;38:217–29. doi: 10.1016/j.gloenvcha.2016.03.010 (PMC4862443; doi:10.1016/j.gloenvcha.2016.03.010)
Supplement: Supplementary file 1 [file mmc1.docx]

**Appendix A.** Three examples of index insurance triggers used in the choice experiment, including (A) prolonged crop inundation, (B) hailstorm, and (C) heavy windstorm causing crop lodging.

| (A) 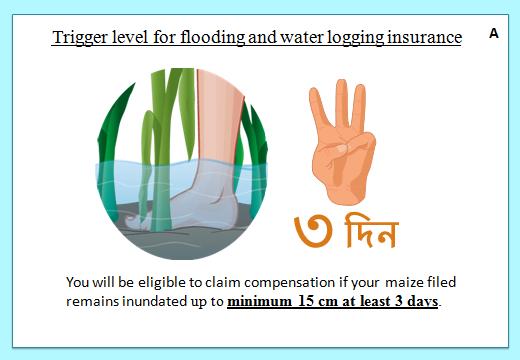 |
| --- |
| (B) 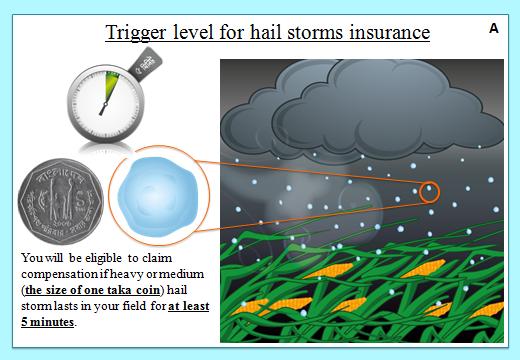 |
| (C)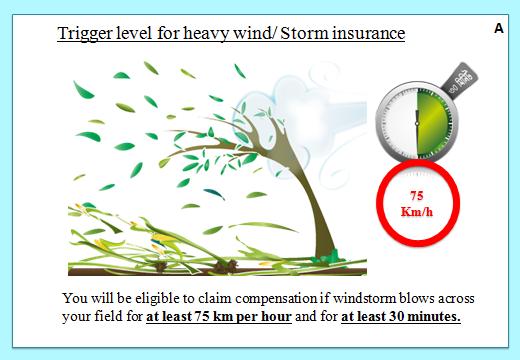 |

**Appendix B.** Description of the insurance scheme presented to the respondents (English translation)

Script for the enumerator to read to the respondent:

_____________________________________________________

Let me first explain how an insurance scheme works even if you are already familiar with it.

- Insurance is a financial mechanism that helps to reduce risk. If you buy an insurance product you get compensated for hazard related losses.
- You might have heard about health or life insurance. Health insurance requires you to pay a risk premium periodically (once a year or once every six months). In return you get reimbursed medical expenses incurred due to certain health problems. Life insurance compensates the insured’s household for sudden or unexpected losses of life of the insured or for the loss of one/multiple limb/s.
- Similar arrangements can be made to cover crop damage losses arising from weather related hazards such as heavy wind, inundation and hailstorm. Such insurance schemes are known as ‘weather insurance’.

I will now describe the general principles of a weather insurance scheme for maize.

If you want to buy the insurance, you will need to:

- Choose a hazard against which you would like to protect your crop.
- Make a deposit in the beginning of the *rabi* (or *boro*) season [the 1^st^ day of *Poush* (December 15)]

In return:

- You will receive a compensation payment on June 15 (the 1^st^ day of *Ashar*) if your chosen hazard takes place.
- Note that the hazards will be defined by pre-specified weather parameter thresholds known as ‘danger level’.
- If the hazard does not take place (in other words the weather parameters lie below the danger level), you may or may not receive any money back. This will depend on the type of scheme you choose.

In the next questions, I will offer you various insurance options for three different hazards:

1. Inundation caused by river flooding or heavy rain
2. Heavy wind
3. Hailstorm

These three hazards have been identified to be most serious for maize after discussions with agronomists and local agricultural extension workers and farmers. These insurance schemes have different danger levels. Danger level refers to the thresholds of weather parameters at which compensations can be claimed. Please take a careful look at the following cards that explain the trigger levels for each of the hazard types.

*Note for the enumerator:*

*Show the cards (see Figure 1 for examples) related to trigger levels one by one. Ask the respondent if he/she clearly understands them. If he/she does not understand, explain again. Do not proceed until the respondent fully understands the trigger levels.*

The payment will be made upon verification of the weather indices.

Verification:

- There will be no assessment of actual crop damage. Your compensation will depend on the verification of the weather parameters.
- Verification of these weather parameters will be done through local weather stations.
- There may not be a weather station in your village or union now. But if insurance is offered in your village in future, a weather station (or a branch station) will be established first for obtaining reliable measures of weather parameters.
- The staff of the village weather station will regularly monitor the weather parameters and visit the fields of the insured farmers.

1. Do you understand the verification process?

0 = No🡪 [enumerator: explain again]

1 = Yes

Availability:

- Your local NGO will be the most likely provider of this insurance scheme.
- The insurance will be available in your village in future only if we identify sufficient demand for it.
- Also, the cost of offering such contracts needs to be compared with the potential revenue they are likely to generate.
- At the moment, we do not know the date when the insurance scheme might become available.

Next, I will show you SIX cards. Each card will present two options (schemes).

The options will vary based on:

- - hazard type
  - the amount of deposit you have to make on December 15 (the 1^st^ day of *Poush*)
  - the amount of money you receive on June 15 (the 1^st^ day of *Ashar*) if your chosen hazard takes place
  - the amount of money you receive on June 15 (the 1^st^ day of *Ashar*) if your chosen hazard does NOT take place

**Appendix C.** Cheap Talk Script (English translation)

“I would like you to choose one option out of these alternatives in a way as if you are making a real purchase decision. Your responses are very important, as they will be used to design a real insurance contract in this region. When you make a choice, I would like you to remember that there are other ways to cope with weather-induced damages. For example, the strategies you have used in the past.”

**Appendix D.** Data Collection (further details)

Enumerators were full- or part-time students from local colleges having no affiliation to any government or non-governmental organizations. Two of the principal investigators provided training in Bangla (4-days) to the enumerators on the survey instruments and how to administer them to the respondents. Trainings involved simulations and pre-tests to assure they were able to correctly administer the surveys in Bangla.

Interviews with the sampled maize farmers were scheduled by mobile phone for all sampled respondents. They were informed that an enumerator would ask detailed questions about farming practices and costs and returns from maize production. If the respondent, particularly the female respondent, was not fully aware of this information, then the presence of the person in charge of farming was requested. To keep track of the extent to which different household members participated in the responses, the enumerators were asked to answer the following questions at the end of the *choice experiment* module:

*Q1. Was any member of the household present during the interview of this segment?*

*Q2. If yes, what is the relationship of the member with the respondent?*

*Q3. Did the respondent consult with the family member?*

*1. Not at all; 2. Sometimes; 3. Most of the time; 4. Always.*

On average, each interview lasted for approximately three hours. Respondents were paid a Tk. 100 (USD 1.30) participation fee which was equivalent to 35% (40%) of the nominal daily wage rate for a male (female) agricultural worker in rural Bangladesh during June 2014.

The questionnaire consisted of approximately 100 questions divided into 12 modules that contained questions about demographic and socio-economic conditions, for example related to living standards (e.g. income, expenditure, land- and non-land assets, etc.). Respondents were also asked about their perceptions of natural hazard risk, crop damage incurred due to natural hazards in the past 10 years, and the nature and extent of any support received from government and NGOs. Risk preference was elicited using the method detailed by Eckel and Grossman (2002). Respondents were asked to choose one out of a list of six gambles that varied both in terms of expected pay-off and uncertainty. Payoffs were determined by a coin toss. The outcome of the gamble was paid to a telephone-mobile bank account nominated by the respondent within 24 to 48 hours after the survey. The maximum pay-off was Tk. 250 (USD 3.25), 12.5% less than the daily agricultural wage. Time preferences were elicited using a consequential question about whether the farmers would prefer to accept the participation fee of Tk. 100 (USD 1.30) immediately, or a higher amount after one month. The maximum payment one could receive by waiting was Tk. 170 (USD 2.20), which was 59.5% of the daily wage rate.

**Appendix E.** Criteria for determining the optimal number of segments

| Number of segments | *N* | Number of parameters (P) | Log likelihood (LL) | pseudo R^2^ | AIC3^a^ | BIC^b^ |
| --- | --- | --- | --- | --- | --- | --- |
| 1^c^ | 433 | 23 | -2457.57 | 0.13 | 4984.14 | 2527.38 |
| 2 | 433 | 33 | -2368.13 | 0.16 | 4835.27 | 2497.71 |
| 3 | 433 | 56 | -2329.40 | 0.17 | 4826.78 | 2549.28 |
| 4 | 433 | 79 | -2298.61 | 0.18 | 4834.21 | 2609.81 |

^a^ AIC3 (Bozdogan AIC) = -2LL+3P

^b^ BIC (Bayesian Information Criterion)=-LL+(P/2)*ln(*N*).

^c^ Random parameter logit model. The coefficients of DEP, Wind×DEP and Hail×DEP were assigned a bounded triangular distribution in which the location parameter is constrained to be equal to its scale so that it does not take non-negative values in the estimation (Hensher and Greene, 2003). The coefficients of the remaining variables were assigned a random distribution. Replication=500.
